# “If only I had”, patients’ experiences during early oncology trials

**DOI:** 10.1007/s00520-023-07738-y

**Published:** 2023-04-21

**Authors:** Maaike E. A. van Sasse van IJsselt, Diane A. J. van der Biessen, Andrea J. van Puffelen, Debbie G. J. Robbrecht, Wendy H. Oldenmenger

**Affiliations:** Erasmus MC Cancer Institute, Department of Medical Oncology, Dr. Molewaterplein 40, 3015 GD, Rotterdam, The Netherlands

**Keywords:** Oncology, Early clinical trials, Experiences, Expectations, Motivations

## Abstract

**Purpose:**

Until today, it is not clear why patients decide to continue with early clinical trial (ECT) participation. Therefore, the aim of this study is to explore to which extent the self-determination theory of Ryan and Deci, according to the ECT enrollment phase, corresponds to the motivations of participants during ECT’s.

**Methods:**

This study has a qualitative design. Data were collected using semistructured interviews and were deductively analyzed in Nvivo12 using the thematic analysis approach of Braun and Clarke.

**Results:**

As a result of the deductive analysis performed, six themes and twenty subthemes emerged which matched the three personal needs: competence, relatedness, and autonomy (*n* = 11). “Competence” included the following themes: mixed future expectations, treatment expectations, and control of the outcome. “Relatedness” included the theme altruistic motivation. “Autonomy” included the themes; to live and act in harmony as well as mental and physical burden.

**Conclusion:**

Participants felt they tried everything and that they were treated to the limit. This not only gives the motivation to continue participating but also a sense of altruism. Despite different burdens, side-effects, and the feeling of being a test subject, the participants will not easily choose to stop participation in order to prevent saying afterwards: “If only I had”.

## Introduction

**Fig. 1:**
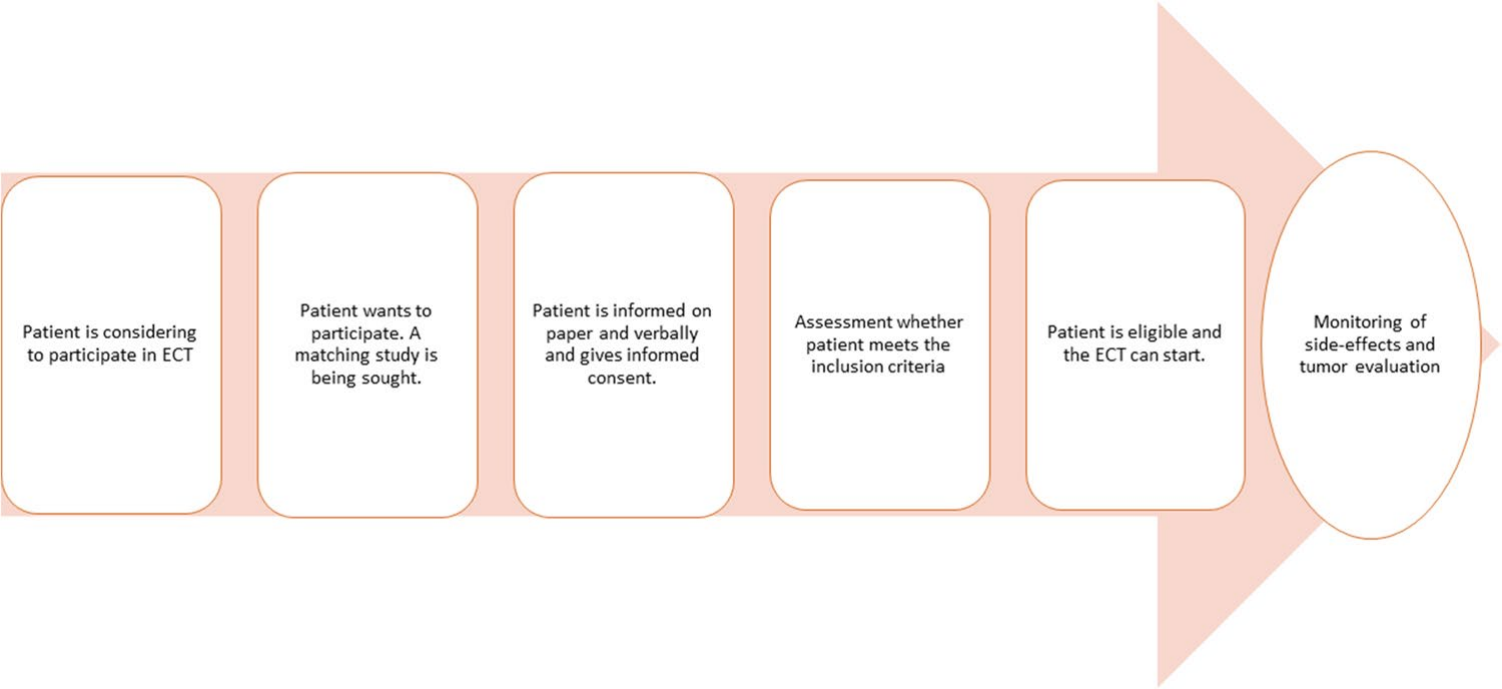
Early clinical trials procedures explained

Cancer is the most common disease in Western Countries. In the Netherlands, the incidence of cancer is one of the highest in Europe [1]. Although treatment options have improved during the last decennia, patients in the palliative setting will be confronted with the fact that standard treatment options for their cancer are no longer available [2]. If this occurs, patients may consider participation in early clinical trials (ECT). ECT evaluates new antitumor compound or new treatment combination in humans [3]. Patients are closely monitored for side effects during ECT (Fig. 1). Evaluation of tolerability and tumor response occurs which will likely result in two possible scenarios; the patient may continue ECT participation, or the patient has to withdraw due to adverse events or progressive disease. Based on this, ECT participation can be divided into two phases, separated by the moment of the first tumor evaluation; participants who recently enrolled and had no tumor evaluation yet (moment 1), and participants who had one or several tumor evaluations and are further along with the trial (moment 2).

On average, two-seven percent of patients with cancer participate in ECT [4]. Consequently, research has been focused on patients’ motivations leading to a decision to participate. Three frequently mentioned concepts are described if it comes to the motivations of patients in the enrollment phase: “Therapeutic optimism”, “Therapeutic misconception”, and “Altruistic motivation” [5, 6]. In addition, Van der Biessen et al. (2018) showed that the motivations of patients leading to a decision to participate can be traced back to the self-determination theory of Ryan and Deci [4, 7]. In this theory, there are three personal needs that influence the motivation behind choice: competence, relatedness, and autonomy [7]. These personal needs can be linked to the frequently mentioned concepts. The first personal need, competence, is interpreted as the way to influence personal outcomes and can be linked to therapeutic optimism and therapeutic misconceptions. Therapeutic optimism is seen in more than 80% of ECT participants. They are hopeful, optimistic, and motivated by the potential of a clinical benefit [4–6, 8, 9]. Although trials are framed by strict scientific standards and are different from registered therapies, 68% of ECT patients had therapeutic misconception [6]. Lack of understanding the nature and purpose of the ECT and the inability to distinguish between the aim of the trial and the actual treatment lead to distorted expectations [5, 8, 10, 11]. Relatedness is the second need and can be understood as a particular manner of connectedness with other people, which may affect decision making. The concept “altruistic motivation” can be linked to relatedness: Patients genuinely want to help researchers obtain scientific knowledge that might benefit future patients with the same disease [5, 6]. Autonomy is the third need and is defined as the condition of self-government to live and act in harmony with one’s integrated self. If a patient lacks autonomy, they can feel controlled by forces that are not in line with who they are [7, 12].

The existing literature gives insights into the motivations of patients why to participate in ECT. However, when patients participate in ECT, a significant decrease in health-related quality of life outcomes and a decrease in hope during trial participation is detected [13–15]. Until today, it is not clear why patients decided to continue with ECT participation [16]. Therefore, the aim of this study is to explore in which extend the self-determination theory of Ryan and Deci according to the enrolment phase corresponds to the motivations of participants during ECT participation. These outcomes may support advanced care planning during trial participation and will lead to a well-informed decision-making process after trial participation [17].

## Methods

### Study design, population, and domain

This study has a deductive qualitative design based on the self-determination theory of Ryan and Deci. Data were collected using semistructured interviews. The study was conducted according to the guidelines of the Declaration of Helsinki and approved by the Institutional Review Board and the local Ethics Committee of the Erasmus MC Rotterdam (MEC-2020-0006). All participants provided written informed consent before the interview.

COREQ guidelines were followed [18]. Participants were eligible for inclusion in this study if they were (1) older than 18 years, (2) participating in oncology ECT at the Centre of Drug Development of the Erasmus MC, and (3) able to speak and understand the Dutch language. A purposive sampling strategy was used to ensure a maximum variation based on cancer diagnosis and moment of participation. Participants who recently enrolled and had no tumor evaluation yet were classified in moment 1 and participants who had one or several tumor evaluations and were further along with the trial were classified in moment 2. Interviews were conducted between February 2020 and July 2021.

### Procedures

The nurse practitioners from the ECT staff identified eligible patients and invited them to participate by phone. If interested, they received verbal and written information. During the following visit, the nurse practitioner discussed participation. After consent, an interview was conducted by the executive researcher in a separate room.

### Data collection

An interview guide was composed to give direction to the interviews. The topics were based on those of the self-determination theory of Ryan and Deci [7], previously mentioned literature and relevant literature [19]. Topic one was motivation which corresponds with competence (therapeutic optimism) and relatedness (altruistic motivation), the second topic was expectations which corresponds with competence (therapeutic misconception), and the last topic was experience which corresponds with autonomy (to live and act in harmony). An example question for topic one was “What are your reasons for continuing participation in ECT?”. The questions were broadly posed and do not steer the participants in any particular direction. The interview guide was pilot-tested before the first interview and evaluated within the ECT clinical team [20]. Notes about remarkable statements or actions and non-verbal communication were written down during and after the interview [21].

### Data analysis

Data had been deductively analyzed in Nvivo 12 using the thematic analysis approach of Braun and Clarke according to the model of Ryan and Deci’s self-determination theory [22]. Analysis occurred within 2 weeks to prevent information loss [23]. The total process of analyzing was an iterative process where constant comparison took place [20]. To enhance the trustworthiness and accuracy of the data, all steps were reviewed. To ensure the completeness of the analysis, all information was seen as important and therefore included in the transcripts and analysis [20]. The first four interviews were conducted in 1 week, and analysis started the following week. First, the transcripts were read several times to become familiar with the data, after which the first codes were generated. These codes were discussed and adjusted by consensus. Hereafter, the codes that matched made initial themes. The analysis was repeated after every two interviews. This resulted in some codes being moved to other themes, merged, or adjusted in consensus until consensus was reached. Data saturation occurred when no new codes and themes emerged in the two final interviews during analysis [22, 24]. This occurred during analysis of the last two interviews, there were no adjustments in codes, and themes were made. All participants were asked for a member check [25]. They agreed and did not report any errors.

## Results

### Participants and demographic data

Eleven patients were willing to participate. The mean duration of the interview was 27 min and 35 s. Additional demographic characteristics and information about diagnosis are shown in Table 1.

**Table 1:** Demographic characteristics of the phase-I participants (*n* = 11)

	Total
Gender	
Female	4 (36%)
Male	7 (64%)
Age	
40–50	2 (18%)
51–60	3 (27%)
61–70	3 (27%)
71–80	3 (27%)
Marital status	
Single	2 (18%)
Married	9 (82%)
Moment of interview^1^	
1	6 (56%)
2	5 (44%)
WHO^2^	
0	3 (27%)
1	8 (73%)
Cancer diagnoses	
Melanoma	1 (9%)
Glioblastoma	1 (9%)
Leukemia	2 (18%)
Prostate cancer	3 (27%)
Endometrial cancer	1 (9%)
Lung cancer	2 (18%)
Ovarian cancer	1 (9%)

^1^(1) Participants who recently enrolled and had no tumor evaluation yet; (2) Participants who had one or several tumor evaluations and are further along in the trial; ^2^WHO = WHO Performance Status

### Findings

**Fig. 2:**
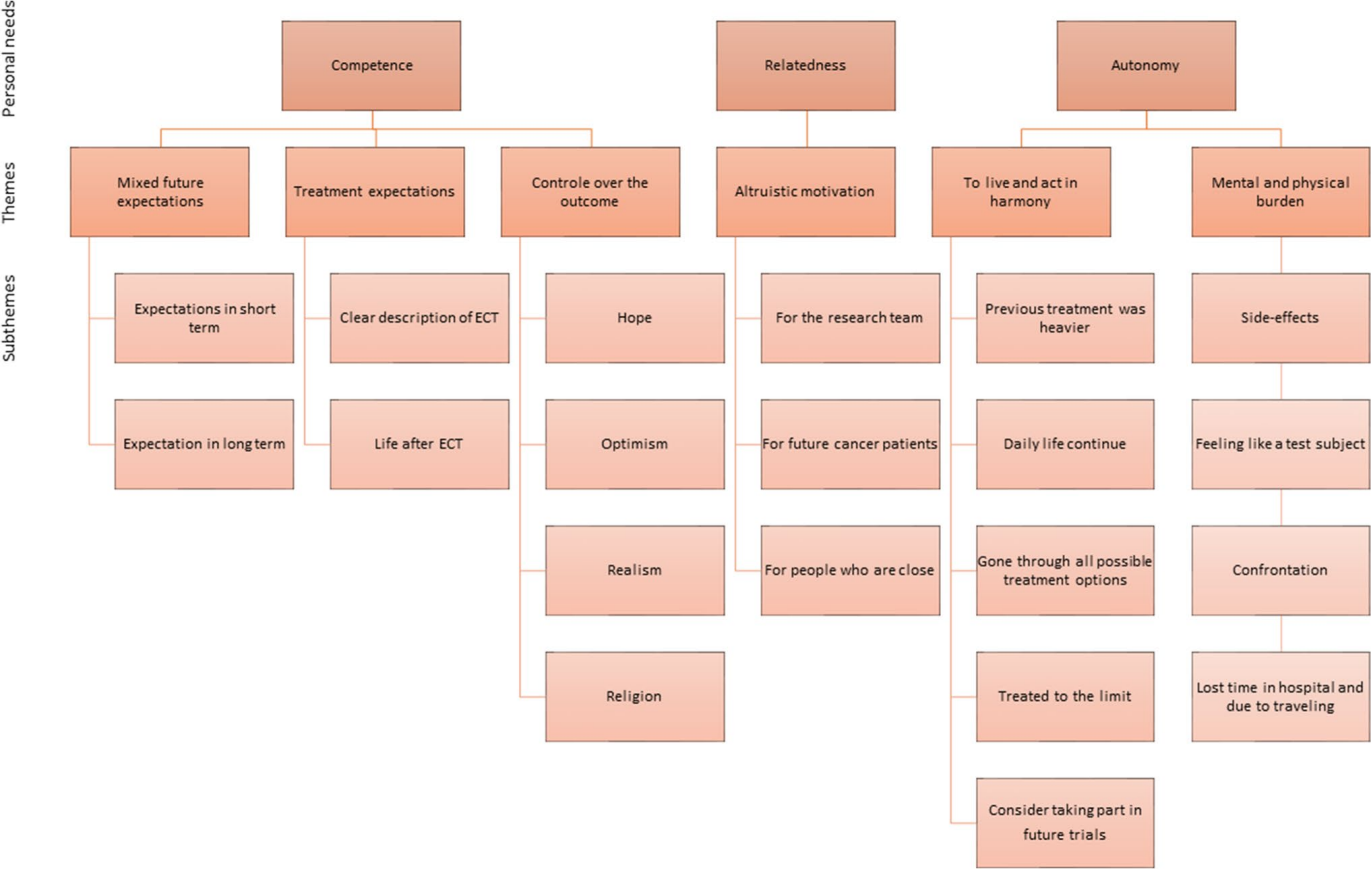
Personal needs, themes, and subthemes

As a result of the deductive analysis performed, six themes and twenty subthemes emerged matching the three personal needs (Fig. 2).

#### Competence

##### Mixed future expectations

The future expectations of the participants varied from short-term expectations (days–week), to long-term expectations (month–year). Prior to the first evaluation, participants tended to look into the future in the short term.

“Now I live by the day. Now I live to arrange everything in such a way that when I die, everything can continue. I was busy with arranging that in the past, but not very intensively. And that has completely changed. My point of view is completely reversed.”

Following the first evaluation, many participants tended to look further into the future.

“My expectation is that I can continue trial participation for a while. That I can continue this trial for a few more years.”

##### Treatment expectations

All participants gave a clear description of ECT, and they all described how their treatment would go according to the trial protocol in detail.

“Especially the fact, you look again at what effect it can have for the rest of your life. You also know when it doesn’t work, then it has no value in the end. You look at the point that it does apply to you. You know, the changes are small.”

Almost all participants thought about life after ECT. The themes which came forward were end-of-life care, such as hospice care and euthanasia. This notion confronted them with the fact that eventually they would have to leave their loved ones behind. A small number of participants indicated that they also needed guidance in this regard.

##### Control of the outcome

Control of the outcome was measured as “hope of living longer”. Participants hoped to participate in a trial which would be life-extending and give as few side-effects as possible. In addition to hope, participants sought motivation in optimism or realism. Optimism was found in the fact that participants were optimistic about the possibility of extending their lives by the maximum amount. They thought that this possibility may apply to them. Participants even said that the probability of success was greater than zero. Participants reasoned that there were two options: they would benefit from participation, or they expected to stay in the same state as before they started, not including the possibility that their health could be worse due to the treatment (e.g., toxicity, burden, impact on quality of life).

“I have more optimism than hope. I need more optimism than hope, I can see that for myself. It is wonderful when hope is included, but I want to stop that cancer, and I want to live in a normal way for a few more years. That is reasonable, that is, not hope. I just want that.... Yes. There is still hope. There is still a chance. And I grab it with both hands. I'll see where it ends. And this makes sure that I don't feel down at the moment. That I am not depressed. I still feel motivated.”

Participants remained as positive as possible; they shared the opinion that without this positive approach they could not have sustained participation. Participants who had recently enrolled in ECT emphasized that the probability that the ECT could extend their lives was small and they were realistic about the outcome and their future perspectives. Only one participant indicated to have received motivation from their religion.

#### Relatedness

##### Altruistic motivation

Some patients wanted to do good for the research team and future patients. Participants saw it as their duty to contribute to the development of new anticancer treatments, from which future patients may benefit. Ultimately, this will help future cancer patients.

“It is important for the people who come after me. I also participate to satisfy the researcher.”

Furthermore, participants wanted to stay alive as long as possible for their family, partners, and friends. Participants’ motivations were influenced both by the opinions and actions of their loved ones and the research team.

“I do it for others and for myself. Seeing your grandchildren grow up. I have been married for almost 58 years. But I would still like to reach 60 years of marriage.”

#### Autonomy

##### To live and act in harmony

A small number of participants said that experiencing as few side-effects as possible with no side-effects was an advantage. If they did have side effects, some compared it with past regular treatment, which they experienced as heavier.

“At some point I could no longer tolerate the chemo. Then we switched to other things that did not work... It was too intense for me. We were halfway through that, and he (the doctor) said: I don't want your death to my conscience.”

Harmony was also found in the fact that daily life, such as their hobbies and work, could be continued. Prior to the first tumor evaluation, participants were just “happy” that they could participate, and participants during ECT participation experienced stabilization or a decrease in cancer progression. This indicated that participants found harmony and peace in the fact that they have gone through all possible treatment options.

“I go along with it and we see how far we get. If I don't do this, I could say: ‘If only I had .. Now I don’t have to say that. Now I have done it all. I have tried everything... The motivation why you do it, I do this to make sure I have tried everything. Without any doubt.”

Some even experienced the feeling that they have been treated to the limit or that they “are not ready yet”.

“It gives me the feeling that I am not ready yet. It is as simple as that.”

All participants would consider consent in the future trials. Furthermore, they indicated that it is not an option to reconsider current trial participation. All participants thought about the quality of their life.

“The way I live right now, it is bearable. Every time I am here for a week and I get all the side effects. So be it. Is still doable for me. If my health deteriorates and I have to come in here in a wheelchair, uhm, things like that. I have an euthanasia statement and then it is over.”

##### Mental and physical burden

Most participants experienced the expected side effects. The participants expressed their concerns according to certain aspects of the trial such as blood sampling, hospital admissions, exact time of intake of medication, amount of medication, and feeling of being a test subject.

“Expectations. They just aren’t there. You’re a kind of. Experiment. It is an experiment… and the protocol that is very fixed.”

During ECT, almost all participants mentioned that the trial confronted them with the fact that there is no regular treatment option available and the fear that they may have had to stop trial participation. Some participants felt burdened by the fact that they had to travel to the hospital and sometimes spend their precious days on a hospital visit.

“For me phase I, this study of mine is the last possible option... It consumes a lot of energy. This creates fatigue again. At least to me. Constantly working on it. I get tired because of that, it almost makes me want to give up life (of trial participation). Physically. And therefore, not getting enough quality of life.”

## Discussion

The three personal needs defining the motivation theory of Ryan and Desi can be found in the emerging themes (Fig. 2). First and most important is the finding that ECT participants do accept the experienced side-effects and the burden of participation in the hope that it will prolong their life. This can be seen as a way to influence their personal outcomes. ECT participants experienced mental and physical burden and the stain of having no other treatment options left. Nevertheless, they consider taking part in future trials. They not only want to control their outcomes, but also want to do good for one another and live in harmony. There is a fine line between hope, optimism, and realism, with continuous shifts between optimism and realism, while they all remain hopeful. Another finding consists of the differences between participants who recently enrolled in an ECT and had no tumor evaluation yet (moment 1) and participants who had one or several tumor evaluations and are further along the trial (moment 2). First, the participants in moment 1 examined the future in the short term, while participants in moment 2 dared to look further into the future. This is consistent with the additional finding that participants in moment 1 did not know what to expect, felt tension, and tended to have more fear of having to stop trial participation.

Our study result show that the first personal need competence not only includes therapeutic optimism. The theme “control of the outcome” contains, in addition to optimism, also hope, realism, and religion. Participants are hopeful for an ECT where the side effects are less or none compared to the previous treatments and which can extend anticipated life expectancy. Literature even shows that 48.7% of 300 patients answered “no” when they were being asked whether the phase I trial could cure their cancer [26]. In addition, participants have a complex relationship between knowing the reality of their situation and hoping that there still might be a treatment that would have a positive effect. “Trying everything” appeared to be a way of maintaining hope [27, 28]. It is this hope that ensures a lower psychological distress score and even a positive relationship was found between hope and perceived health [26–28]. Unexpectedly, these study results partly agree with the concept of therapeutic optimism in the enrolling phase. All participants talked about their treatment expectations in accurate detail; they were well informed about the nature and purpose of the trial. This expresses the change in future expectations associated with either moment 1 or 2. This finding can be related to the fact that most patients also participated in another ongoing study towards a better communication about choices in palliative care and ECT participation [29]. Lastly, mixed future expectations were also a theme that can be classified under personal need competence. While some participants indicate that they thought carefully about their quality of life and about the moment they want to stop the phase I trial, they want some more guidance in this process. Not discussing posttrial care gives the feeling that there is no path forward, which is followed by distress [30].

The second personal need is relatedness, which can be linked to the theme “altruistic motivation”. The participants genuinely want to help researchers obtain scientific knowledge that might benefit future patients with the same disease. The observation of altruism matches findings in other studies [5, 6, 8, 28]. This approach is not surprising, as the literature shows that the closer patients are to death, the greater the desire to help others [27].

The third and last personal need is autonomy; the desire to be in charge of one’s own decision to live and act in harmony with one’s integrated self. Two themes are classified under this personal need: to live and act in harmony and mental and physical burden. A new finding is that participants seem to mitigate the expected side effects, feeling that they are a test subject, the confrontation with death, and the loss of time by ECT by putting them into perspective and comparing them with the previous (more heavier) treatment(s), the fact that they are still able to perform daily activities, that they have tried all possible treatment options and that they are being treated to the limit. As a result, despite this mental and physical burden, they reconsider consent in the future. This new finding corresponds to the fact that patients who are in the process of stopping ECT, still hope that there will be another treatment that will cause them to avoid palliative care [13, 17].

A major strength of this study is the deductive qualitative design with the use of the constant comparative method and reaching saturation during data collection [17, 31]. A possible reason to reach saturation after only eleven interviews might be the selected patient group that participates in ECT. In addition, the internal validity is improved by discussing the findings until consensus was reached. Recall bias were reduced by interviewing participants during their ongoing phase I trial participation and by analyzing the interviews within 2 weeks [23]. A limitation of this study is that it was only performed at one university hospital. Hereby, the generalizability of the study might be reduced. Nevertheless, this study included participants from different studies, with different types of cancer and variation in age.

A recommendation based on these study findings is that it is meaningful to recognize and acknowledge the mental and physical burden of participants. The previous treatment, continuing daily life activities, going through all possible options and being treated to the limit, put these burdens into perspective. Besides this, it is important to reflect on the guidance of these patients that in addition to hope, there is also optimism, realism, and religion that influence their motivation. Lastly, in order to give patients better support during ECT and support advanced care planning, it is recommended to pay attention to whether the patient is participating in moment 1 or 2. This way, the future perspective can be anticipated and a well-informed decision about life in and after ECT can be made.

It can be concluded that participating in ECT is a great uncertainty. Participating creates the feeling that ECT patients are trying everything and are treated to the limit. This not only gives the motivation to continue participating but also a sense of altruism. Family and friends, hope, realism, optimism, and helping to develop a new drug also provide motivation. Despite different burdens, side-effects, and the feeling of being a test subject, the participants will not easily choose to stop participation in order to prevent to say afterwards: "If only I had".

## Data Availability

Available on request
